# Involving service users in the qualitative analysis of patient narratives to support healthcare quality improvement

**DOI:** 10.1186/s40900-018-0133-z

**Published:** 2019-01-03

**Authors:** Louise Locock, Susan Kirkpatrick, Lucy Brading, Gordon Sturmey, Jocelyn Cornwell, Neil Churchill, Glenn Robert

**Affiliations:** 1 Health Services Research Unit, University of Aberdeen (Formerly University of Oxford), Health Sciences Building, Foresterhill, Aberdeen, AB25 2ZD Scotland; 2 Health Experiences Research Group, Nuffield Department of Primary Care Health Sciences, University of Oxford, Woodstock Rd, Oxford, OX2 6GG England; 3 Institute of Psychology Health and Society/North West Hub for Trials Methodology Research, University of Liverpool, Liverpool, L69 3GL England; 4 Aberdeen, Scotland; 5 Point of Care Foundation, 3rd Floor, CAN Mezzanine 7–14 Great Dover Street London SE1 4RY, London, England; 6 Experience, Participation and Equalities, NHS England, Quarry House, Quarry Hill, Leeds, LS2 7UE England; 7 Florence Nightingale Faculty of Nursing, Midwifery and Palliative Care, King’s College London, James Clerk Maxwell Building, 57 Waterloo Rd, London, SE1 8WA England

**Keywords:** Patient and public involvement, User involvement, Patient experience, Experience-based co-design, Qualitative analysis, Qualitative interviews, Quality improvement, Health research

## Abstract

**Plain English summary:**

Patient or user involvement in health research is well-established but is often limited to advising on research questions and design, leaving researchers to collect and analyse ‘data’ (which in this paper means written copies of interviews with patients about their experiences). We were working with sets of interviews with 1) young people with depression and 2) people with experiences of stroke. We were looking for key themes that it would be useful for the NHS to know about, and we developed short films which healthcare staff can use to think about how to make care more patient-centred. We wanted to see what user involvement in this analysis would bring, and how best to achieve it practically.

After the researcher team had analysed the interviews, we ran two one-day workshops with people with relevant experience as a patient/service user or carer. We gave them some brief training in how to analyse interviews and how they might be used for improving the quality of care. Then we looked at extracts from the interviews, and discussed whether people could see the same themes as the researcher.

People identified similar themes to the researcher, but also identified new details the researcher had missed. However, they felt reading large amounts of text was not the best way to use their time and experience. Instead they recommended that a better approach would be for a researcher to meet with a group of users at the start of analysis, to discuss what to look out for.

**Abstract:**

**Background**

Patient or user involvement in health research is a well-established principle. However, involvement is often limited to advising on research questions and design, leaving researchers to complete data collection and analysis. Involvement in data analysis is one of the most challenging, least well-explored aspects of involvement. Qualitative interview data forms high volumes of rich, complex material which can be daunting to work with.

Analysing narrative interviews with patients is central to a patient-centred quality improvement method called experience-based co-design. The analysis identifies ‘touchpoints’ – key moments of healthcare experiences – and leads to the production of a ‘trigger film’ to spark codesign discussions between patients and staff. We wanted to see what user involvement in this analysis would bring, and how best to achieve it.

**Methods**

As part of a wider secondary analysis study to create new trigger films, we re-analysed interview transcripts on experiences of young people with depression and experiences of stroke. We then ran two workshops with people with relevant lived experience, working with extracts from the same materials after brief training.

**Results**

People involved in the workshops identified similar themes to the researcher, but also brought some new insights. While they engaged easily with the materials selected, we under-estimated how much time it would take people to work through these. Discussion and sharing experiences and perspectives were highly valued in the first workshop. In the second workshop, we therefore started with group discussion, based on people’s own experience, of what they thought the touchpoints would be, and later viewed a draft trigger film together to see how it compared.

**Conclusions**

Those involved felt that while analysing transcripts was possible in small quantities, it was not best use of their time. We suggest that conversation, rather than data, is at the heart of user involvement in analysis. One way to retain the value of lived experience in the analytic process, without over-burdening people with data, is to elicit user reflections on their experience at the start of analysis, and use this as a guide to direct both researcher and service user attention during the remainder of the process.

## Background

### User-centred narratives and quality improvement

Patient experience is defined as one of three components of quality in healthcare, alongside patient safety and clinical effectiveness. Improving people’s experience of healthcare has been highlighted as a priority for successive governments in the United Kingdom [1]. A number of specifically user-centred quality improvement approaches drawing on patient experience data have been developed, such as Experience-Based Co-Design (EBCD) and Patient and Family Centred Care [2–4].

Despite a substantial amount of evidence about what matters to people about their care, until recently organisational initiatives have commonly focused on collecting more patient experience data (typically through surveys) and measuring performance, rather than using it for quality improvement [5]. This situation is gradually changing, but clinical staff keen to use patient experience data for improvement may lack organisational support to do so. They need expert help sifting through such data to identify themes and priorities for action, and implementing quality improvement [6, 7]. This may be particularly true of narrative data. As Martin et al. [8] note, making sense of quantitative data is challenging enough; working out how to turn the ‘untamed richness’ of ‘soft’ forms of intelligence such as narrative and observational data into action for quality improvement may be even more daunting.

Bringing service user partners into the analysis process may be one way to help achieve this. We involved users in a secondary data analysis project which aimed to develop new resources to support EBCD.

### Patient and public involvement and data analysis

Patient and public involvement (PPI) in health research is a well-established principle, meaning research is conducted with or by users, rather than to, for or about them. Service-user-researchers, who collect and sometimes analyse research data, are more common in some research fields than others, notably mental health. However, more typically PPI involvement has been limited to advising on research questions and research design, leaving professional researchers to complete data collection and analysis. PPI in data analysis is perhaps one of the most challenging and least well explored aspects of involvement. Analysing patient stories may appear more intuitive and approachable than, say, quantitative analysis such as logistic regression or mathematical modelling. At the same time, both the volume of the data generated and the need for the analyst to be alert to theory as well as patterns in the data can be a hurdle.

There are comparatively few articles documenting the process of and rationale for service user involvement in data analysis. A systematic review [9] found many examples of lay people becoming involved in the conduct, design or dissemination of research, but it was less common for them to be involved in execution or translation of the research. This echoes findings that ‘examples are few of participatory interpretation and analysis of data’ [10]. There are however some interesting examples of service user involvement in the analytic process, which focus both on the process of ‘doing qualitative analysis’ with lay partners, but also offer insights into the benefits and challenges. These challenges include how much training is required and to what level of detail; practical constraints and demands of reading through long transcripts; how to recruit user partners able to engage with the perspectives of the specific study population; and how to deal with the emotional impact of reading potentially difficult stories [11–13].

Whitmore [14] cautions against expecting lay partners to write for an academic audience (which could be ‘unfair and unrealistic’), and distinguishes this from involvement in interpretation and illuminating the meaning of participants’ accounts. The knowledge and understanding that lay partners bring to the table has been argued to offer clear benefits, and to counter the interests, assumptions and perspectives which researchers themselves bring to analysis.

Jennings et al. [15] have recently reviewed different approaches to ‘collaborative data analysis’ within mental health and propose the following typology:

- Consultation (researchers conduct the analysis and present it to PPI partners for comment)
- Development (PPI partners help develop an initial coding framework then applied by the researchers)
- Application (researchers develop a coding framework and then involve PPI partners in applying this to a set of transcripts)
- Development and application (in which PPI co-researchers are given extensive training in data analysis, and are involved over time in both developing and refining the coding framework and applying it to all data, described as the ‘gold standard’ of collaborative data analysis).

The authors note that while studies using ‘consultation’ may appear to be the least democratic approach, they also ‘tended to have people with lived experience in their research team….[and] moved beyond the binary categorisation of researchers as academic or service users’ [15 p.4]. One example used ‘multiple coding’, with one clinical researcher, one psychologist and one service user researcher each independently coding focus group transcripts to identify outcomes for Cognitive Behavioural Therapy for psychosis [16]. The results showed a high degree or consensus between the analysts but also new themes, and ‘points of fracture or non-consensus’ (p.e96) which had to be resolved, in some cases by returning to individual research participants to clarify what they had meant.

An example of the gold standard ‘development and application’ approach is Cotterell [17]. To support his study of the needs of service users with life limiting conditions he held repeated interpretation and ‘theme generation’ sessions over the course of several months with a user panel. The researcher’s initial seven themes were revised to eight themes, only two of which (‘diagnosis’ and ‘relationships’) remained the same. Strikingly the revised themes included more emotional categories than the original set, including ‘fear’, ‘anger/frustration’, and ‘grief’. Garfield et al. [18] describe a lighter touch process of involving lay partners in analysis of qualitative data, by giving people a sub-set of transcripts and inviting them to come up with their own themes after brief training. These mapped closely onto the themes already identified in the researcher-led analysis, providing useful confirmation, but also identified one new theme. The final output of the research was presented as a ‘synergy of perspectives’ that were not easily visible or separated.

Best et al. [19] tested the Participatory Theme Elicitation or PTE method, with a youth advisory panel of 8 members. The research team selected 40 focus group data extracts, from a study of school-based physical activity, that ‘could be easily understood and interpreted as standalone statements’ (p.3). The young people involved were invited to sort these into thematic piles. These were then recorded and grouped by researchers, and the output was then compared with the researchers’ thematic analysis. Echoing Garfield [18], the authors found that ‘while PTE analysis was, for the most part, consistent with the researcher-led analysis, young people also identified new emerging thematic content’ (p.1). They note that approaches to involving people in analysis need to be accessible, and that some techniques (such as Byrne et al’s [10] experimentation with the Voice-Centred Relational or VCR Method) are too elaborate and time-consuming in terms of training and analysis time. Byrne et al. themselves identify that teenagers involved in the VCR method - which involves repeated attentive reading of transcripts and the development of an emerging narrative -found it ‘difficult, tedious and time consuming’ p.74).

Some researchers who have involved users in analysis have expressed concerns about lay partners potentially putting too much emphasis onto their own experiences rather than analysing what came out of the data [18]. Cotterell [17], however, records that his early concerns about ‘the ability of the group to remain ‘objective’ and to refrain from blurring interpretation of data with their own personal experience and concerns’ proved unfounded. He concludes that service users with similar concerns and experiences as those who are the focus of the study can bring a reflective lens to the process and a greater capacity for the analysis to truly ‘unpack the taken for granted’. One might add that researchers also bring their own biases about what they see in the data and what they regard as important.

Fisher [20] argues that involvement of lay partners in analysis can uncover nuances which may not be obvious to a researcher. Disabled user researchers drew his attention to a single passage in one transcript which for them gave an important sense of how users feel judged and oppressed by benefit assessment panels, but to which he might not otherwise have given much weight. This demonstrated how insider sensitivity to the nuance of language can bring a level of understanding to the process that researchers may not attain, even after long immersion within a community. Similarly, Gillard et al. [21] suggest that service user involvement in data analysis added ‘expertise by experience’ to the process, and had changed the conventions of ‘doing research’ to some degree by the inclusion of challenging voices, as well as adding a more nuanced set of themes that came from their differing points of reference.

### What is experience-based co-design (EBCD)?

EBCD is a participatory action research approach to quality improvement based on active patient-staff partnership [2]. It has three phases: ‘discovery’, ‘co-design’, and implementation.

The discovery phase, which would normally last around 6 months, involves interviews with staff; observations of a particular care pathway; and video-recorded interviews with local service users and family members about their experiences of care. These interviews are analysed by a researcher (who may be an academic or a healthcare professional) to identify ‘touchpoints’ – these are key moments of interaction between person and service where something could have been done better, or which exemplify good experience. Video clips showing these touchpoints are selected and edited into a ‘trigger film’. Examples of touchpoints might include an account of insensitive communication; a memorable act of kindness or emotional care; disturbance from noise or lighting on the ward; particular efforts to ensure privacy and dignity.

In the co-design phase, the trigger film is shown first to a workshop for users and family members only, and then again to a joint workshop with healthcare staff. The aim of the film is to trigger discussion about local quality issues and agree a set of improvement priorities, which are then addressed through small co-design working groups (involving users and staff as equal partners). The whole EBCD process typically takes 12–18 months to complete [22].

Although evaluations have shown EBCD to be effective, they have also identified the time and resources it takes as barriers to widespread adoption [22]. In a previous study [23]we therefore tested an ‘accelerated’ version of EBCD in two lung cancer services and two intensive care units, in which we replaced much of the discovery phase with interviews from existing national interview collections, held by the Health Experiences Research Group, University of Oxford [2] and disseminated on the Healthtalk.org website through a series of lay-friendly ‘topic summaries’. Thus instead of trigger films made from local user interviews, we identified touchpoints from existing interviews which had been collected around the country, and made films which can be used in multiple locations. However, in common with other EBCD projects, this did not include direct user involvement in analysis.

### Rationale for the new project

Having demonstrated that trigger films developed from the national archive could have a similar effect to local films, and were ‘good enough’ to trigger co-design, we were prompted to reflect whether further adaptations to the discovery phase were possible. The co-authors were awarded a grant for secondary data analysis from the Economic and Social Research Council (ES/L01338X/1) to make a series of new trigger films and pursue two possible ways to streamline the discovery phase:

1. What would be lost, if anything, if touchpoints for the trigger films were selected only from material already chosen for dissemination on Healthtalk, rather than re-analysing full transcripts, as a way of further accelerating the process (i.e. from the topic summaries on the website)?
2. How could service users themselves best get involved in data analysis? Would they identify similar or different touchpoints to the researcher?

In this paper we focus particularly on this second area, and describe the work we undertook with user partners in analysis workshops. We conclude with recommendations developed with our workshop attendees for taking the process forward and our reflections on it.

As well as having a service user co-investigator on the team [GS], we organised two workshops to explore involvement in data analysis, with two service user panels; one panel member has also co-authored this paper [LB].

## Methods

### Initial researcher-led analysis

Secondary analysis [24] was undertaken on two existing HERG interview collections: ‘Experiences of stroke’, and ‘Experiences of young people with depression’. These topics were among several selected for re-analysis in consultation with our research partner at NHS England (NC) as national priorities. We focused on these two topics for user involvement because they offered diversity in terms of both the age groups affected and a mix of mental and physical conditions. Re-analysis of both the original transcripts for each study (n = approximately 40 for each study), and the summarised topic sections on the healthtalk website, was first undertaken by a researcher [SK]. This focused on identifying ‘touchpoints’ of care (described above), coding for narrative extracts that demonstrated ways in which service experiences impacted either positively or negatively on people’s overall experience of care, using a framework analysis [25, 26].

### User analysis workshops

We then sought to sense-check the analytic themes identified by the researcher in two workshop sessions with users. The aim of the workshops was to help us understand more fully to what extent service users might become involved in the development of trigger films and to consider how this might bring new insights to the process of data analysis for trigger film development. We also anticipated that the workshops would help us to think more broadly about the practical feasibility of involvement at this level, gaining feedback from those involved on how they felt about taking part in such activities, and whether they might suggest other ways of working.

Researchers SK AND LL approached groups and organisations associated with people with lived experience of both health conditions (young people with depression, and stroke), using a flyer and poster. People interested were provided with details of how to contact the team and discuss any questions before deciding whether they would like to be involved. Arrangements for each workshop were made to fit with the needs of each group, involving a short day (approximately 10 am to 3 pm) of activities associated with qualitative data analysis, including refreshments, lunch and social time. Everyone was paid for their time and travelling expenses.

At the end of both workshops, we asked people for any verbal feedback on the process of being involved which could help us improve the process in future, and some people also sent us further feedback by email afterwards.

The format originally for the workshops included a brief training introduction to qualitative analytical methods; an explanation of EBCD, trigger films and the concept of touchpoint;, followed by participants reading interview transcript extracts and website summaries to identify touchpoints. Workshops were facilitated by the Principal Invesitgator (LL) and lead researcher (SK), with support from a research assistant. Each is described below.

### Workshop one; young people’s experiences of mental health services

This workshop aimed to work on the development of a trigger film on young people’s experiences of mental health services. Six young people (ages 19–23) agreed to be involved. Four were identified from the INVOLVE young people’s mental health advisory panel; one had been a research participant in a previous healthtalk project about antidepressant use; and one had experience of a previous EBCD project on a different health topic. The workshop was also attended by our service user co-applicant who provided the group with insights from his own experiences of working with EBCD projects, and co-author GR. Workshops were not recorded but SK and LL were present throughout and the research assistant took notes Table 1.

**Table 1:** Workshop 1 agenda

1. *Introducing qualitative research – informative session about the Healthtalk website, how data is collected, and how it is used*	
2. *About our project – how Healthtalk data can be used for EBCD work*	
3. *Informative session explaining EBCD and how it works in practice*	
4. *Being involved in a co-design project – Workshop participant talking about involvement in a previous EBCD project on chemotherapy*	
5. *General group discussion about the project, questions, and sharing experiences*	
*Lunch Break*	
6. *Group discussion about ‘touchpoints’*	
7. *Informative session introducing data analysis*	
8. *Hands-on workshop - looking at interview transcripts and website data.*	

### Workshop 2 – Experience of stroke, or caring for someone who had had a stroke

This second workshop aimed to work with service users on the development of a trigger film about experiences of stroke. For this workshop, users were identified through a regional PPI involvement network, using a poster and flyer advertising the workshop. The poster was also displayed on community notice boards in several locations. Four people who had experienced a stroke, and one person caring for a relative who had a stroke agreed to get involved. They were all over 50 years of age. The note-taker assistant was also available to help workshop attendees navigate through the paperwork. This was important to ensure that people with impairments resulting from their stroke could feel comfortable taking part. As previously SK and LL were also present throughout. The structure of the session was adapted from that of workshop 1, taking account of the learning we had gained from that session (see ‘Results’ below), as well as to accommodate specific needs of the client group Table 2.

**Table 2:** Workshop 2 Agenda

1. *Introducing qualitative research – how we collect data for the healthtalk website, and how it is used.*	
2. *Our project – how healthtalk data can be used for EBCD work.*	
3. *EBCD – how it works in practice*	
4. *Workshop attendees’ own examples of touchpoints*	
5. *Introducing data analysis*	
Lunch Break	
6. *Hands-on workshop- finding ‘touchpoints’ in topic summaries from the healthtalk website.*	
7. *Review draft trigger film material*	
8. *Comments and discussion*	

For this workshop SK and LL provided similar but shorter presentations to the group about qualitative research, healthtalk and EBCD. Next, instead of analysing interview transcripts, the group were asked to identify what they thought of as key touchpoints for stroke care, based on their own experiences. We then asked them to review a website ‘topic summary’ on stroke experiences, again looking for touchpoints. Towards the end of the session we asked the group to watch a draft half-hour film that had been pre-prepared by SK after completing her independent analysis. We asked them to consider how well it encapsulated the touchpoints they had identified earlier in the day.

## Results

### Workshop 1 – Young people and depression

Our reflections on workshop 1 were that both the informative sessions in the morning and the subsequent analysis work in the afternoon took far longer than we had anticipated. Having prepared four transcript extracts and five topic summaries, we only managed to go through one transcript extract fully (working independently first and then comparing page by page as a group) and most of a topic summary (explored collectively page by page). However, given that this was a workshop designed specifically to explore the feasibility of these activities, it provided us with interesting insights into aspects of the process including whether or not the tasks appeal, or are practical to engage with, for people with differing interests and capabilities.

Analysing transcripts for quality improvement purposes involves reading through a lot of material that may not be relevant in order to identify touchpoints as well as deciding in each case whether a particular passage could be viewed as a touchpoint, and if so, why. In the group, a lot of time was spent reflecting on this, for each of the extracts that were identified. Some feedback suggested it was difficult to interpret a touchpoint because of this way they are interwoven with other aspects of the person’s narrative, making selecting and focusing a very difficult task. For example, a brief comment about access to services may be embedded within a longer narrative of early symptoms and inner feelings. Maybe researchers are so used to analysis, abstraction, summarising, and illustrating that we underestimate how complex this might be for others, despite the advantage of lived experience they bring.

The group were very focused on the importance of early intervention for depression. This was something SK had identified from her own analysis of the transcripts, and was therefore not an entirely new theme. However, the group’s input was powerful in terms of the emphasis and insight that came from their own lived experience, which in turn reinforced the importance of this aspect of service provision for the research team. This then influenced the selection of touchpoints on this topic for the trigger film.

Engaging with the material (both transcripts and summaries from the website) stimulated interesting discussions amongst the group about their own experiences, one key reason why we got through less material than originally planned. Although at first we viewed this as a failure on our part to ‘keep things on task’, it became clear that the value was in this very aspect – in giving space for the young people to discuss how their own experiences compared with the interview data. The impulse to discuss each touchpoint in detail also provided some personal benefit of sharing stories and mutual support – which one young person identified in her feedback as one of the main attractions of doing involvement activities.

Feedback suggested that although initially the idea of a touchpoint was difficult to grasp, it became clearer as they applied it to sections of text and agreeing examples. Group discussion and working in pairs helped shed more light than solo-working on what to look for and how to read the transcript in different ways. Facilitated discussion helped the young people refine and clarify their ideas, and gain confidence in sharing their own opinions and experiences. It also felt more enjoyable and less like an ‘exam scenario’, as one young person put it. There was general agreement that the research team’s expectations of how much material we might get through was unrealistic, and that we needed to reduce the volume considerably for any future analysis workshops.

Some of the group of young people were keen to get involved in further work. One (co-author LB) wanted to take on extra analysis to do later at home, and eventually decided to follow a career in research (see Table 3). Another young person who had not been able to attend the workshop also worked on some transcript material at home.

**Table 3:** Co-author LB reflections on workshop 1

I felt very engaged in the workshop and was keen to get involved and help develop the EBCD quality improvement process. It was empowering to use my own experiences, which at times had been bad, for something positive. The workshop started with an introduction to qualitative research and touchpoints. As a group we worked through the first few examples, and I felt able to identify touchpoints. After working through some of the transcripts independently, we compared what we had found and there were a lot of similarities. During the workshop, I identified a series of touchpoints which resonated with my own experience. As a group, we discussed these parallels, and I found this therapeutic. I enjoyed coding the transcripts and at the end of workshop asked if I could have more to continue working through at home. The workshop, and working with the research team, led me to a change in direction. After this introduction to qualitative research, I decided this was something I was really passionate about, and I wanted to work in this field. I am now doing a PhD exploring PPI in the development of core outcome sets.	

Of course not everyone who gets involved in research as a user will end up training as a researcher, but this account resonates with a wider literature on the personal benefits of involvement, for example Thompson et al’s [27] suggestion that it might ‘offer spaces for the reconfiguration of identity’ (p.47).

### Workshop 2 – Stroke

Based on our experience of Workshop 1, the length and detail of the introductory sessions on qualitative analysis and experience-based co-design were considerably shortened for Workshop 2. In addition, rather than starting the analysis work going through transcript extracts, we invited the group to discuss together what they thought might be likely touchpoints for stroke case, based on personal experiences. This generated a rich discussion and a list of candidate touchpoints. Examples included:

- Professionalism/caring attitude from staff - nursing, but also e.g. x-ray staff
- Staff being respectful regardless of patient attitude – emotions and difficult feelings can manifest in different ways, but some patients are difficult and demanding
- Being given strategies for coping. Knowing what helps and being able to explain it to others – communication
- Importance of information and explanations – patients may need repeated explanations due to cognitive impairments as well as emotional problems
- Noise, light, alarms going off
- Importance of being offered physiotherapy, what happens when it is not available
- Lack of long term support after discharge
- Unsettling to be in ward with dementia patients
- Delays in referral for rehabilitation appointments
- Long waiting times
- Need more information about what to expect and how to deal with things e.g. personality changes, emotions, sensory overload

We then asked them to review a website ‘topic summary’ on stroke experiences, again looking for touchpoints. We found their assessment produced very similar results to the researcher perspective, though given varying degrees of fatigue as well as visual and cognitive impairment some found dealing with written material less easy or engaging than the verbal touchpoint discussion.

Towards the end of the session we asked the group to watch a draft half-hour film that had been pre-prepared by the researcher after completing an independent analysis. This had been based around the following themes:

**Table:** 

Theme	Issues covered under this theme
Having a stroke	Importance of swift help; symptom recognition; ambulance care
Hospital care and communication with health professionals	Delays on arrival at hospital; importance of kindness and understanding; staff assumptions, and poor communication; feeling blamed or judged; contrasting different staff groups; being treated as a person; culturally insensitive care; lack of information about what was happening; involving family members
Personal care	Dignity in personal care; staff recognising the emotional impact of having to be washed; help to become independent in personal care;
Rehabilitation	Importance of goal-setting; practical advice and support of therapy professions for mobility, swallowing, speech; ambivalence about support groups; rehab needing to be maintained over a longer period
Transition to home	Practical advice on managing in the home environment; need for more ongoing support; difficult for family too; community support when living alone
Dealing with emotions	Anxiety; need for psychological support and treatment; family emotions; loss of identity
Final thoughts	Listen to the patient as a person

Overall, the group felt the film reflected well the themes relating to stroke services that they had brought up, but with some differences in both content and emphasis; for example, noise and light disturbance, sensory overload and disturbance from other patients were themes they identified which did not feature in the draft film. As well as the content of the film, the group commented that it was important for clips to be audible and succinct; they would have preferred a shorter film.

We invited feedback about the workshop process. Despite efforts to reduce the amount of information-giving about the analysis process in response to feedback from the first workshop, they felt that there had still been too much to get through in the early informative sessions so that after lunch they were starting to feel tired. Some also felt that the information had been too dense to engage with. One suggestion they gave to simplify the process was perhaps to ask people to come to the session with a short list of things that mattered to them the most about stroke care, and/or potentially sending out materials to them to work on at home, at a pace and time that they could control.

However, there was general support for the value of a group discussion to pool ideas about touchpoints as an accessible way to contribute. There was consensus that this made better and more efficient use of their time and knowledge than text-based work, and could be a way forward for collaborative analysis.

Everyone involved said they had enjoyed the day and that it felt good to be involved in something with the potential to make a difference. Two expressed a sense of frustration at no longer being able to work due to their strokes, and that taking part in this type of activity helped them to feel a sense of self-worth and being valued.

The trigger films they worked on, along with others produced by researcher-only secondary analysis, are now publicly available at http://www.healthtalk.org/peoples-experiences/improving-health-care/trigger-films-service-improvement/topics.

## Discussion and Recommendations

Working with service users to help think about how best they might become involved with devising trigger films using the HERG interview collection was a learning process for both us and our user partners. As other research teams before us have found, user involvement in data analysis poses a number of practical issues. In summary, the two groups of service users who worked with us reported that they found the process interesting and engaging, and they came to understand what to look for in the data, although it took some longer than others to reach that point and some found the process tiring and difficult to engage with. They identified very similar themes and touchpoints to those that the researcher had selected in the data samples they worked with, but also through general discussion of their own experiences. Some additional themes or differences in emphasis were identified.

The nature of the analytic task in this case (identifying topics relevant for quality improvement, from existing interview collections) was fairly practical and applied, and did not require specific expertise in sociological theory or analysis. We felt some understanding of the process of data collection and analysis would be helpful, as well as explaining EBCD and the concept of ‘touchpoints’. However, despite our efforts to reduce the amount of training in qualitative research methods between the first and second workshops, we still did not get this right. In future we would focus less on general qualitative analysis methods. Our impulse was to think that methods training was essential to reduce the power differential between researchers and PPI partners, and equip them to deal with large amounts of text. Yet training is only empowering if it is targeted and useful; we were not clear enough about what was needed and why. With hindsight, training people to be more like researchers and process lots of text was probably misplaced, in this case.

In working with transcripts, we came to realise that even the small sample of data we had hoped to get through was too ambitious in the time available. In the wider secondary analysis study, we found that for the purposes of identifying touchpoints for a trigger film, working with summarised findings on healthtalk was ‘good enough’ and even we as researchers did not really need to go back to full transcripts. Thus working only with these summarised findings might have been a better option in our PPI workshops.

Given that even experienced qualitative researchers can feel overwhelmed and puzzled by how to make sense of mounds of data in what is often a lengthy and immersive process of analysis, we should not expect service users to feel any less daunted. People with cognitive impairment or fatigue related to their condition can find concentrating for long periods especially challenging, as reported by those who had had a stroke. Even without health difficulties, some people understandably just find reading large amounts of material difficult, boring or hard to get to grips with, as some young people in this project reported. Equally, reading repeated accounts of other people’s experiences can be tiring or upsetting, although in this project no-one reported being distressed.

Conversely, there can be benefits for people, for example providing an opportunity for them to ‘give something back’ and contribute to improving services. It can also offer a space to meet people and discuss their own experiences with others in a supportive environment [27], although this in itself needs to be carefully managed. Our service user co-author (GS) who had been involved in a previous EBCD project recalled that in that project some people found recalling their experiences upsetting (though they had been well supported by a clinical nurse specialist and chose to continue being involved)*.* However, he felt that the workshops for the current project seemed to offer a secure and supportive environment, where sharing lived experience felt positive and purposive rather than exposing.

Co-author LB, who went on after the project to be awarded a PhD studentship for a qualitative study of PPI, demonstrates that some users can get deeply involved, but her experience and level of interest are probably atypical. It would not be appropriate for involvement in analysis to be confined only to those willing to pursue such an interest to this level. However, it does indicate wider benefits of PPI in building capacity for individuals and the NHS.

One reason why data analysis took even longer than we anticipated was because reading transcript material often prompted users to compare and contrast this with their own experiences, and to generate lengthy discussion with the researchers and other service users at the workshop. This has sometimes been identified as a ‘problem’ with PPI, as in some way a deflection from the task in hand. But as other researchers have found, analysis by people with lived experience can bring new insights that might be missed by analysts with more professional concerns, and it is important to allow space for these reflective conversations, rather than artificially steer people back to a pre-set agenda.

From discussion with the people who got involved in this work, we have come to the view that conversation rather than data is at the heart of user involvement in analysis. We suggest that one way to retain the value of lived experience in the analytic process, without over-burdening people with training, time and ‘mounds’ of data, is to elicit user reflections at the start of analysis, and use this as a guide to direct the researcher’s gaze. In this we are drawing on Blumer’s notion of the ‘sensitising concept’ to ‘suggest directions along which to look’ [28]. Later in the process of trigger film development, key themes from the data analysis can then be presented for feedback and validation, to check whether the themes adequately reflect those identified by users at the outset, whether anything is missing and whether changes in emphasis are required. This sits more within the ‘development’ approach identified by Jennings et al. [15] than ‘development and application’, though in our case having a lay co-investigator [GS] and a co-researcher keen on ‘application’ who became a co-author [LB] somewhat blurs this distinction.

The Jennings et al. best practice framework [15] was not available to us when working on this project, but is designed to support collaborative data analysis in a wide range of qualitative study types. They note particularly that applying the ‘gold standard’ or ‘development and application’ is often not feasible in time and resource-limited studies. Instead they propose that the research team (which should include people with lived experience) should develop a preliminary coding framework, share it with a PPI co-researcher group through a workshop to coproduce a refined coding framework, which the research team then apply to the data and reflect on the results with the PPI co-researcher group. What we have conceptualised as an analytic conversation early in the analysis phase bears remarkable similarity to this framework.

We conclude that the contribution of lay partners in the analysis of data can add a valuable layer to the process, ensuring the priorities of service users are firmly at the forefront of the analysis. Whilst we found much in common between the researcher-led analysis and the themes users felt were important, young people drew our attention to the importance they attach to early intervention, and people with stroke identified some aspects of their inpatient experience such as noise and light disturbance, sensory overload and disturbance from other patients. Our own reflections on the process led us to consider widening the definition of ‘analysis’ in this context, to include early conversation and guidance on the expected and eventual content of the analysis, thus firmly directing the researcher gaze to prioritise service user lived experience. Just as there has been a shift to involve people before any research is done in setting research questions and priorities, so involvement in analysis can equally be at an earlier stage. Even if – as we found – comparisons between what researchers and users see in the data are often more about nuance and emphasis than major differences, these nuances are important.

Madden and Speed [29] argue against a ‘narrow, technocratic co-option of PPI’; instead, involvement should be about.

‘populations engaging in the decisions that impact their lives, identifying opportunities and strategies for action. Being “critically involved” requires acknowledging processes of situated contestation rather than epistemic authority, identifying varieties of publics and the contingency and complexity of the construction of evidence’ (p.5).

As researchers, we discovered we were too attached to our own process, our own concern with methods, and to the idea that analysis inevitably means close immersion in large amounts of text. But users told us this was not realistic, nor was it best use of their time and insights; they preferred conversational engagement in the analytic process. They saw it as their role to guide us – in effect to equip us with a map and a compass - but not to try to become qualitative analysts labouring over multiple transcripts.

Thus user involvement in analysis as it has evolved for us can be a more creative, more discursive, less process-driven approach, breaking out of researcher norms of both what constitutes analysis and who can do it. This resonates with previous findings that one of the ways in which user partners can redress the power balance and create a more equal conversation with researchers is through exercising a form of symbolic capital as ‘challenging outsider’, maintaining a distinctive non-researcher perspective [30].

Finally we note Staley et al’s [31] suggestion that the impact of involvement should not be conceptualised narrowly as changes to specific tasks or steps in a single research project, but rather as a more diffuse ‘learning experience’. They argue for a shift of emphasis from how involvement changes research towards how it changes researchers themselves, and their ‘values, preferences and practice’ (p.6). The analytic conversation may change the way we think, not just about a specific transcript or project, but about how we approach the analysis process more generally in the future.

## Abbreviations

EBCD: Experience-based co-design
NHS: National Health Service
NICE: National Institute for Health and Clinical Excellence
PPI: Patient and public involvement
